# Efficacy and safety of esaxerenone (CS-3150) for the treatment of essential hypertension: a phase 2 randomized, placebo-controlled, double-blind study

**DOI:** 10.1038/s41371-019-0207-x

**Published:** 2019-05-21

**Authors:** Sadayoshi Ito, Hiroshi Itoh, Hiromi Rakugi, Yasuyuki Okuda, Satoru Yamakawa

**Affiliations:** 1 Division of Nephrology, Endocrinology and Vascular Medicine, Department of Medicine, Tohoku University School of Medicine, Sendai, Japan; 2 Division of Endocrinology, Metabolism and Nephrology, Department of Internal Medicine, Keio University School of Medicine, Tokyo, Japan; 3 Department of Geriatric and General Medicine, Osaka University Graduate School of Medicine, Suita, Japan; 4 Daiichi Sankyo Co., Ltd., Tokyo, Japan

**Keywords:** Medical research, Health care

## Abstract

This was a phase 2, multicenter, randomized, double-blind, placebo-controlled, open-label comparator study to investigate the efficacy and safety of esaxerenone (CS-3150), a novel non-steroidal mineralocorticoid receptor blocker, in Japanese patients with essential hypertension. Eligible patients (*n* = 426) received esaxerenone (1.25, 2.5, or 5 mg/day), placebo, or eplerenone (50–100 mg/day) for 12 weeks. The primary efficacy endpoint was the change from baseline in sitting systolic and diastolic blood pressure (BP). Safety endpoints included adverse events and serum K^+^ elevation. There were significant dose–response reductions in the 2.5 and 5 mg/day esaxerenone groups for sitting BP (both *p* < 0.001) and 24-h BP (both *p* < 0.0001) compared with placebo, with a mean (95% confidence interval) change in sitting BP of −7.0 (−9.5 to −4.6)/−3.8 (−5.2 to −2.4) mmHg in the placebo group, and −10.7 (−13.2 to −8.2)/−5.0 (−6.4 to −3.6) mmHg, −14.3 (−16.8 to −11.9)/−7.6 (−9.1 to −6.2) mmHg, and −20.6 (−23.0 to −18.2)/ −10.4 (−11.8 to −9.0) mmHg for the 1.25, 2.5, and 5 mg/day esaxerenone groups, respectively, while the change was −17.4 (−19.9 to −15.0)/−8.5 (−9.9 to −7.1) mmHg for eplerenone. The incidence of adverse events was similar in all treatment groups. Serum K^+^ levels initially increased in proportion with esaxerenone dose but were stable from week 2 until week 12. Plasma esaxerenone concentration increased in proportion with the dose. In conclusion, esaxerenone is an effective and tolerable treatment option for patients with essential hypertension.

## Introduction

Hypertension is a multifactorial disease involving complex interactions between various metabolic, neurohormonal, and inflammatory factors, and is a leading risk factor for cardiovascular morbidity and mortality [1, 2]. Uncontrolled hypertension is associated with vasculopathy, heart disease, cerebrovascular diseases, and nephropathy, all of which are classic manifestations of hypertensive end-organ damage [3].

In addition to non-pharmacological treatments, management of hypertension often requires pharmacotherapy with an antihypertensive agent, which is a proven approach for reducing cardiovascular morbidity and mortality [4]. Although monotherapy may be effective in some patients, failure to achieve the desired antihypertensive effect requires the concurrent use of multiple antihypertensive drugs as part of a multifactorial strategy [5, 6]. In fact, many patients require three or more antihypertensive drugs to achieve a blood pressure (BP) level of <140/90 mmHg [7]. Triple drug therapy combinations usually include a renin–angiotensin system inhibitor (angiotensin converting enzyme [ACE] inhibitors or angiotensin II-receptor blockers), a calcium channel blocker, and a diuretic.

The Japanese Guidelines for the Management of Hypertension (2014) recommend adding an aldosterone antagonist to the treatment regimen of patients with poorly controlled BP or resistant hypertension [8]. Aldosterone is a steroid hormone that regulates electrolyte homeostasis and BP via binding to the mineralocorticoid receptors (MR) in the distal tubule and collecting duct of the kidney [9]. Primary aldosteronism (PA) is caused by the excess production of aldosterone and is the most common cause of secondary hypertension as well as a common cause of antihypertensive treatment resistance [10–12]. Furthermore, aldosterone-induced MR activation impairs insulin sensitivity and is associated with obesity and diabetes [13, 14]. Therefore, treatment-resistant hypertension in the presence of conditions such as obesity, diabetes, and chronic kidney disease (CKD) can occur despite normal plasma aldosterone concentrations (PAC). The role of pathological overstimulation of MR in the absence of high aldosterone levels has been indicated in cases of MR-associated hypertension [15].

Various studies have demonstrated the utility of steroidal MR antagonists, such as spironolactone and eplerenone, in the treatment of resistant hypertension [16–18]. When added to a renin–angiotensin system (RAS) blocker, steroidal MR antagonists further reduce proteinuria in patients with CKD from either diabetic or non-diabetic causes [19]. Therefore, adding an aldosterone antagonist to the treatment regimen of patients with poorly controlled BP, or treatment-resistant hypertension, is recommended in the Japanese Guidelines for the Management of Hypertension (2014) [8], the American College of Cardiology/American Heart Association 2017 guideline for high blood pressure in adults [20], and the 2018 European Society of Cardiology and the European Society of Hypertension guidelines for the management of arterial hypertension [21]. However, spironolactone has significant treatment-emergent side effects such as gynecomastia [8]. While eplerenone has an improved MR selectivity, it seems to be less potent when compared with spironolactone [22]. In general, MR antagonists elevate serum K^+^ levels and, as such, eplerenone is contraindicated in diabetic patients with albuminuria [23].

Esaxerenone (CS−3150) is a novel oral, non-steroidal, selective MR blocker, which is highly potent and could be used to treat hypertension and cardiovascular and renal disorders [24–26]. In preclinical studies, esaxerenone inhibited BP elevation in deoxycorticosterone acetate/salt-induced hypertensive rats and in Dahl salt-sensitive hypertensive rats with an additional protective effect on the heart and kidneys [24, 25]. In a phase 1 study, the tolerability of esaxerenone was confirmed after single- and multiple-dose escalations in healthy Japanese subjects [26]. Therefore, this phase 2 study was designed to evaluate the antihypertensive efficacy and safety of esaxerenone, and to determine the optimal dose for lowering BP in Japanese patients with essential hypertension.

## Patients and methods

### Ethics

The study protocol was reviewed and approved by the independent institutional review board for each center. This study was conducted in adherence with the International Conference on Harmonization Guidelines for Good Clinical Practices, applicable local regulations, and the ethical principles based on the Declaration of Helsinki. All patients provided written informed consent prior to participation.

### Patients

Patients were eligible for enrollment based on the key inclusion criteria: aged ≥ 20 years at time of informed consent; sitting systolic BP (SBP) of ≥ 140 to < 180 mmHg and diastolic BP (DBP) ≥ 90 to < 110 mmHg; and 24-h BP by ambulatory BP monitoring (ABPM) of ≥ 130/80 mmHg. The main exclusion criteria were secondary hypertension or malignant hypertension; diabetes with albuminuria; serum K^+^ level < 3.5 or ≥ 5.1 mEq/L; and creatinine-adjusted estimated glomerular filtration rate (eGFRcreat) < 60 mL/min/1.73m^2^. Additional criteria are detailed in Supplementary Information 1.

### Study design

This was a phase 2, multicenter, randomized, double-blind, placebo-controlled, open-label comparator study across 19 sites in Japan from January to September 2015. All patients underwent a 4-week screening period to remove the effects of prior therapeutic agents. During this period, patients received two placebo tablets administered orally once daily after breakfast.

Eligible patients were then randomized in a 1:1:1:1:1 ratio using stratification by baseline sitting SBP (< 160, ≥ 160) to the following treatment groups: 1.25 mg/day esaxerenone (one 1.25-mg esaxerenone and one placebo tablet), 2.5 mg/day esaxerenone (two 1.25-mg esaxerenone tablets), or 5 mg/day esaxerenone (two 2.5-mg esaxerenone tablets), eplerenone or placebo (two placebo tablets) for 12 weeks. In the eplerenone group, patients were administered 50 mg/day eplerenone for 2–4 weeks followed by 100 mg/day of eplerenone for 8–10 weeks (one 50-mg tablet and two 50-mg eplerenone tablets, respectively).

Esaxerenone and the open-label comparator eplerenone were administered orally once daily after breakfast for 12 weeks. The treatment period was succeeded by a 2-week follow-up period.

The criteria for withdrawal included: the presence of hyperkalemia defined as a serum K^+^ level of ≥ 6.0 mEq/L or ≥ 5.5 mEq/L on two consecutive measurements; SBP and DBP persistently < 90 mmHg and < 50 mmHg, respectively; SBP and DBP persistently ≥ 180 mmHg and ≥ 110 mmHg, respectively; and eGFRcreat values of < 45 mL/min/1.73 m^2^. These criteria were judged by an investigator or sub-investigator to determine whether the patient was to discontinue treatment.

### Prior and concomitant medications

Prohibited concomitant drugs and therapies that could not be used from 4 weeks prior to the start of the treatment period to the completion of the study included antihypertensive drugs (angiotensin II receptor blockers, calcium channel blockers, ACE inhibitors, beta blockers [including alpha–beta blockers], alpha blockers, other sympatholytics, vasodilators, and renin inhibitors); diuretics (thiazide diuretics, thiazide analog diuretics, loop diuretics, and potassium-sparing diuretics); Chinese herbal medicines; cytochrome P450 3A4 inhibitors (itraconazole, ritonavir, nelfinavir mesilate, clarithromycin, and verapamil hydrochloride); K^+^ preparations; and ion exchange resin.

Non-steroidal anti-inflammatory drugs were also prohibited from 4 weeks prior to the start of the treatment period to the completion of the study. However, they could be used for a maximum of 5 days in a row, as external preparations intended for a topical effect (except for suppositories), and aspirin could be used continuously at a daily dosage of ≤324 mg.

### BP measurements

Sitting BP was measured at start, week 3 and the end of the observation period, at weeks 1, 2, 4, 6, 8, 10, and 12 of the treatment period, 1 day after the end of treatment, or at study discontinuation using an automatic BP monitor (HEM-759P Fuzzy device, Omron Healthcare Co., Ltd., Mukou, Japan). Each measurement was taken within 21–27 h after the study drug was administered. BP measurement was always performed prior to blood sampling when both were scheduled on the same day, and at least 3 h after a meal. For each time point, BP measurement was repeated three times with 1- to 2-min intervals following a resting period of at least 5 min while sitting.

In addition, 24-h BP was measured at week 3 of the observation period and week 12 of the treatment period using an ambulatory BP monitor (TM-2433, A & D Co., Ltd., Tokyo, Japan). The study drug was administered after sitting BP measurements and prior to ABPM. BP measurements were taken over a period of at least 25 h with 30-min intervals. The automatic BP measurements and ABPM were operated by the doctors, nurses, or clinical laboratory technicians involved in this study.

### Efficacy analysis

The primary efficacy endpoint was the change from baseline in sitting BP (SBP and DBP) at the end of the treatment period defined as the average sitting BP of week 10 and week 12 after last observation carried forward (LOCF) imputation of missing values. The sitting SBP and DBP (at trough) were measured at baseline and then on weeks 1, 2, 4, 6, 8, 10, and 12. The secondary efficacy endpoint was the change from baseline in mean 24-h BP at week 12.

Other efficacy endpoints included change in sitting BP, change in diurnal, morning (06:00–08:59), daytime (07:00–21:59), and nocturnal (22:00–06:59) BP, the proportion of patients that achieved the sitting BP target (<140/90 mmHg), and the proportion of patients that achieved a mean 24-h BP of <140/90 mmHg.

Subgroup analyses were performed to evaluate the antihypertensive effect (measured by sitting BP) of esaxerenone based on the following baseline factors: sitting BP (SBP and DBP), hypertension grade (Grade I or II) [8], PAC, plasma renin activity (PRA), eGFRcreat, serum K^+^, and the presence or absence of diabetes.

### Safety analysis

Safety variables included adverse events (AEs), clinical laboratory tests (hematology and serum biochemistry), vital signs (sitting BP, pulse rate, and ABPM), body weight, 12-lead electrocardiogram, and serum K^+^ changes from baseline. Clinical laboratory tests and 12-lead electrocardiogram were performed at baseline and on weeks 4, 8, and 12 during the treatment period. Vital signs (except ABPM) were measured at baseline and on weeks 1, 2, 4, 6, 8, 10, 12, and the day after the week 12 visit. Body weight was measured at baseline and the day after the week 12 visit. In addition, serum K^+^ was measured on weeks 1, 2, 4, 6, 8, 10, and 12, and 2 weeks after the end of the treatment.

All the details of AEs and side effects of the study drug have been reported to the drug manufacturer, Daiichi Sankyo Co., Ltd., in the clinical study report document (reference number: CS3150-A-J203).

### Pharmacokinetic analysis

Plasma esaxerenone trough concentrations (C_trough_) were measured from blood samples collected on weeks 4 and 12. The method for blood sample analysis has been described previously [26].

### Analysis of PAC and PRA

The renin–angiotensin–aldosterone system (RAAS) hormones, including PAC and PRA. PAC was measured using a radioimmunoassay and PRA was measured using an enzyme immune assay on blood samples collected on weeks 4 and 12. The methods used to measure PAC and PRA have been described previously [26].

### Statistical analyses

The efficacy analysis was conducted in the full analysis set (FAS), containing patients who were treated with the study drug at least once and for whom measurements were taken for at least one variable pertaining to efficacy after the start of treatment. Safety was assessed in the safety analysis set (SAS), comprising all patients who received at least one dose of the study drug.

The primary efficacy analysis was the change in sitting BP from baseline and was calculated for each treatment group (least squares [LS] mean) with corresponding 95% confidence intervals (CIs) using an analysis of covariance (ANCOVA) model, with change from baseline at end of treatment as the objective variable, treatment group as the explanatory variable, and baseline BP as the covariate. The same ANCOVA model was also used to compare each esaxerenone dosing group with the placebo group. The difference in LS means, corresponding 95% CIs, and *P* values were also calculated. To adjust the multiplicity of statistical tests, the fixed sequence procedure was applied: the comparison was initially conducted between the higher-dose esaxerenone groups and the placebo group with two-sided 5% significance levels, the comparison for the lower dosage groups was continued at a 5% significance level in descending order of dosage but only when significance was shown in the higher dose.

The analysis performed on the primary efficacy endpoint was repeated for the difference in 24-h BP measurements at screening period week 3 and treatment period week 12.

For the remaining efficacy endpoints (change in sitting BP at each visit, diurnal and nocturnal BP [systolic, diastolic, and mean]), summary statistics were calculated by treatment group for each time point and change from baseline. The same ANCOVA model as the primary endpoint was also used to calculate point estimates of change and corresponding 95% CIs by treatment group for each measured time point and to plot trend graphs. For the subgroup analyses, the same statistical model for the primary efficacy endpoint was applied to all subgroups based on sitting BP (SBP/DBP).

Summary statistics were calculated for PAC and PRA by treatment group for each time point. The adjusted LS geometric mean and corresponding 95% CIs were calculated for percentage change in each group at each time point using an ANCOVA model, with change from baseline at each time point an objective variable, treatment group an explanatory variable, and screening period data. Summary statistics were also calculated for determining the pharmacokinetics of plasma esaxerenone concentrations at each time point at weeks 4 and 12. Safety analyses were conducted in a descriptive manner and presented with the appropriate summary statistics by treatment group. All statistical analyses were performed using SAS 9.3 (SAS Institute, Cary, NC).

## Results

### Patient disposition

Of the 687 patients who provided written consent, 426 met the inclusion criteria and were randomly assigned to one of the study groups: placebo (*n* = 87), esaxerenone 1.25 mg/day (*n* = 83), esaxerenone 2.5 mg/day (*n* = 84), esaxerenone 5 mg/day (*n* = 88), and eplerenone (*n* = 84). In the eplerenone group, treatment began at 50 mg/day and was increased to 100 mg/day in 78.6% (66/84 patients) of patients by week 2 and 89.3% (75/84 patients) of patients by week 4. After week 4, no further dose changes were made in the eplerenone group.

**Fig. 1:**
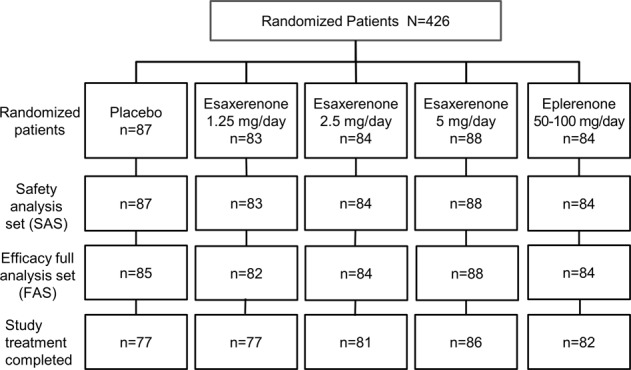
Patient disposition

All 426 randomized patients were included in the SAS, 423 had efficacy data and were included in the FAS, and 403 patients completed the study (Fig. 1). Of the 23 patients who discontinued the study, 13 withdrew by their own choice, four because of AEs, one was lost to follow-up, four met the criterion for withdrawal (both SBP and DBP persistently ≥180 mmHg and ≥110 mmHg, respectively), and one was suspended by the investigators due to high BP.

Patient demographics are shown in Table 1. In brief, 69.7% of patients were male, the mean age was 57.0 years, sitting SBP/DBP at baseline was 157.0/97.6 mmHg, and 52.2% of patients had received prior treatment for hypertension. There were no remarkable differences between treatment groups.

**Table 1:** Patient demographics (full analysis set)

	Placebo *n* = 85	Esaxerenone 1.25 mg/day *n* = 82	Esaxerenone 2.5 mg/day *n* = 84	Esaxerenone 5 mg/day *n* = 88	Eplerenone 50–100 mg/day *n* = 84	All *N* = 423
Sex (male), *n* (%)	60 (70.6)	55 (67.1)	54 (64.3)	65 (73.9)	61 (72.6)	295 (69.7)
Age (years)	57.3 ± 9.1	57.2 ± 9.3	56.8 ± 9.4	57.1 ± 8.8	56.5 ± 10.0	57.0 ± 9.3
Weight (kg)	69.0 ± 13.5	68.8 ± 12.3	67.9 ± 12.1	69.8 ± 13.2	70.7 ± 17.0	69.3 ± 13.7
BMI (kg/m^2^)	25.5 ± 4.1	25.3 ± 3.7	24.9 ± 3.3	25.7 ± 3.7	25.8 ± 4.9	25.5 ± 4.0
SBP (sitting, mmHg)	156.7 ± 9.0	156.4 ± 9.1	156.4 ± 8.4	157.4 ± 9.0	157.9 ± 8.4	157.0 ± 8.8
DBP (sitting, mmHg)	96.8 ± 5.0	97.2 ± 5.5	98.6 ± 5.6	97.2 ± 5.4	98.4 ± 5.3	97.6 ± 5.4
SBP (ABPM, mmHg)	167.0 ± 12.1	166.2 ± 14.7	165.0 ± 15.5	167.1 ± 15.3	165.9 ± 14.0	166.2 ± 14.3
DBP (ABPM, mmHg)	97.9 ± 7.6	98.9 ± 9.0	98.9 ± 10.0	98.5 ± 7.2	98.3 ± 8.0	98.5 ± 8.3
Pulse rate (bpm)	72.1 ± 9.6	73.3 ± 10.2	73.3 ± 9.5	71.7 ± 9.2	73.8 ± 10.2	72.8 ± 9.7
Prior treatment for hypertension^a^, *n* (%)	44 (51.8)	43 (52.4)	43 (51.2)	50 (56.8)	41 (48.8)	221 (52.2)
Presence of diabetes, *n* (%)	9 (10.6)	11 (13.4)	8 (9.5)	10 (11.4)	20 (23.8)	58 (13.7)
LDL cholesterol (mg/dL)	129.1 ± 32.6	127.5 ± 31.0	130.4 ± 31.3	132.0 ± 34.4	127.2 ± 30.2	129.3 ± 31.8
Serum K^+^ (mEq/L)	4.14 ± 0.31	4.07 ± 0.28	4.10 ± 0.25	4.14 ± 0.29	4.09 ± 0.28	4.11 ± 0.29
HbA1c (%)	5.64 ± 0.59	5.67 ± 0.65	5.62 ± 0.63	5.46 ± 0.42	5.76 ± 0.63	5.63 ± 0.60
FPG (mg/dL)	106.3 ± 16.4	109.7 ± 21.2	105.9 ± 17.3	104.2 ± 14.0	109.5 ± 19.9	107.1 ± 17.9
eGFRcreat (mL/min/1.73 m^2^)	78.0 ± 11.6	77.0 ± 12.2	80.3 ± 11.9	79.6 ± 11.5	81.3 ± 12.2	79.2 ± 11.9
PRA (ng/mL/h)	1.05 ± 0.91	1.10 ± 1.05	1.11 ± 1.00	0.96 ± 1.09	1.09 ± 0.97	1.06 ± 1.00
PAC (pg/mL)	116.5 ± 50.15	112.8 ± 35.68	110.0 ± 37.32	107.5 ± 42.51	113.7 ± 39.85	112.1 ± 41.37
Alcohol consumption, *n* (%)						
Never	20 (23.5)	24 (29.3)	23 (27.4)	21 (23.9)	25 (29.8)	113 (26.7)
Former	7 (8.2)	6 (7.3)	3 (3.6)	6 (6.8)	2 (2.4)	24 (5.7)
Current	58 (68.2)	52 (63.4)	58 (69.0)	61 (69.3)	57 (67.9)	286 (67.6)

Data are presented as mean ± SD, unless otherwise stated

*ABPM* ambulatory BP monitoring, *DBP* diastolic blood pressure, *eGFRcreat* estimated glomerular filtration rate with creatinine, *FPG* fasting plasma glucose, *HbA1c* hemoglobin A1c, *LDL* low-density lipoprotein, *PRA* plasma renin activity, *PAC* plasma aldosterone concentration, *SBP* systolic blood pressure

^a^Within 4 weeks prior to run-in period

### Efficacy analysis

**Fig. 2:**
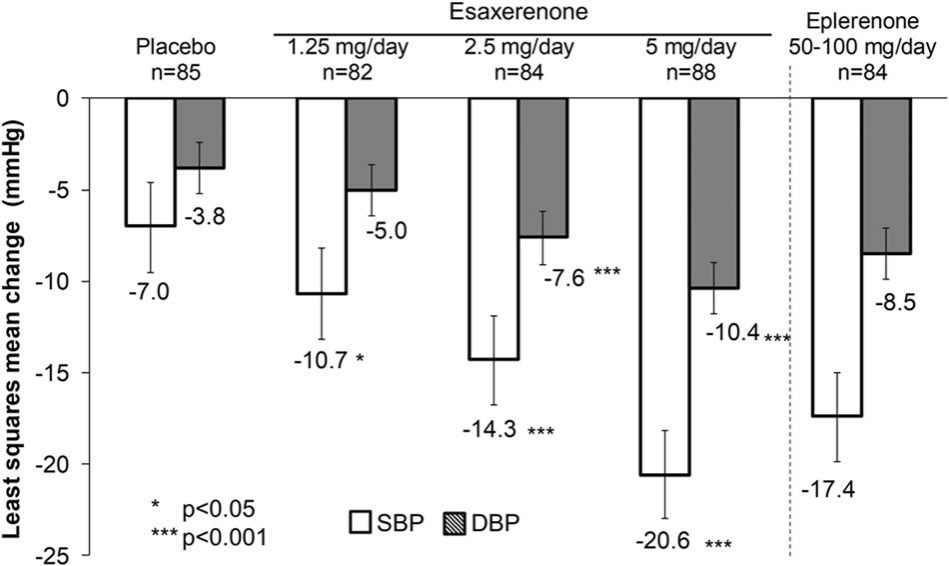
Least square mean change from baseline in sitting systolic and diastolic blood pressures (full analysis set). SBP systolic blood pressure, DBP diastolic blood pressure

**Fig. 3:**
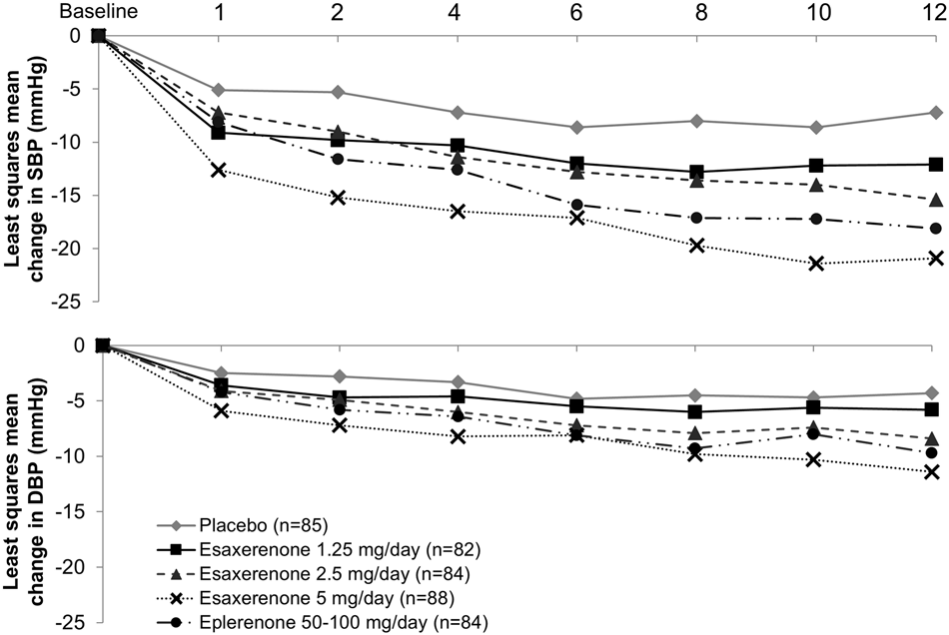
Least square mean change from baseline in sitting systolic and diastolic blood pressures until the end of treatment (full analysis set). SBP systolic blood pressure, DBP diastolic blood pressure

The mean changes from baseline in sitting BP at the end of treatment are shown in Figs 2 and 3. There was a clear dose–response relationship for BP reduction. ANCOVA showed significant reductions in sitting SBP and DBP in the 2.5 mg/day and 5 mg/day esaxerenone groups compared with placebo (all *p* < 0.001). At the end of the study, the LS mean (with 95% CI) change in BP (LOCF) was −7.0 (−9.5 to −4.6)/−3.8 (−5.2 to −2.4) mmHg in the placebo group, and −10.7 (−13.2 to −8.2)/−5.0 (−6.4 to −3.6) mmHg, −14.3 (−16.8 to −11.9)/−7.6 (−9.1 to −6.2) mmHg, and −20.6 (−23.0 to −18.2)/ −10.4 (−11.8 to −9.0) mmHg for the 1.25, 2.5, and 5 mg/day esaxerenone groups, respectively. In comparison, eplerenone therapy produced a BP change of −17.4 (−19.9 to −15.0)/−8.5 (−9.9 to −7.1) mmHg after 12 weeks.

**Fig. 4:**
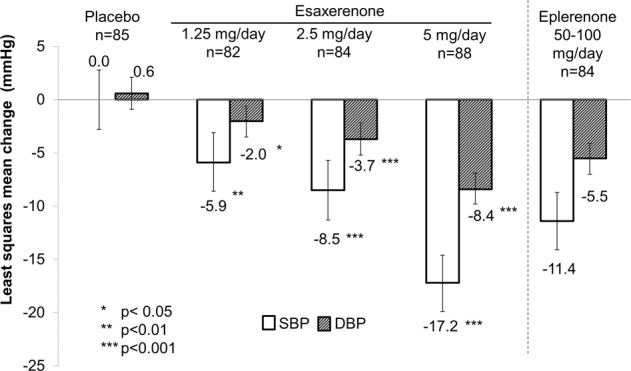
Least square mean change from baseline in 24-h average ambulatory blood pressure (full analysis set). SBP systolic blood pressure, DBP diastolic blood pressure

Similarly, 24-h BP changes showed a clear dose–response relationship, and all esaxerenone doses significantly lowered 24-h BP compared with placebo (1.25 mg/day: *p* = 0.0038 and *p* = 0.0154 for 24-h SBP and DBP, respectively; 2.5 and 5 mg/day: all *p* < 0.0001) (Fig. 4). The LS mean changes from baseline in morning, daytime, and nocturnal BP were greater in the esaxerenone groups compared with placebo (Supplementary Table 1). In addition, the LS mean changes from baseline in morning, daytime, and nocturnal BP after eplerenone treatment were comparable between the 2.5 mg/day esaxerenone and 5 mg/day esaxerenone groups (data not shown).

**Fig. 5:**
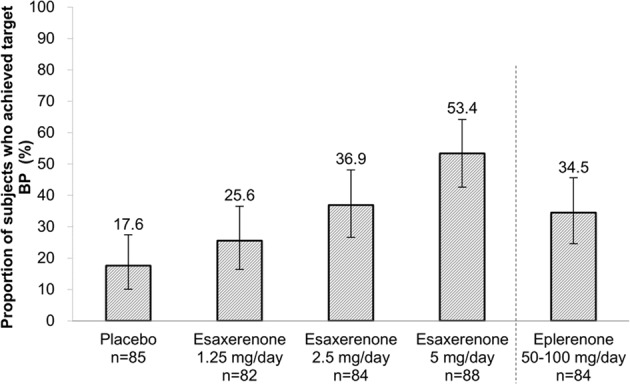
Achievement rate of the target systolic and diastolic blood pressures (full analysis set). Target BP: both SBP < 140 mmHg and DBP < 90 mmHg. SBP systolic blood pressure, DBP diastolic blood pressure

The proportions of patients achieving a target BP of <140/90 mmHg at the end of esaxerenone treatment (LOCF) were 25.6%, 36.9%, and 53.4% for the 1.25, 2.5, and 5 mg/day groups, respectively (Fig. 5). In comparison, the proportions of patients achieving the same target BP were 17.6% in the placebo group and 34.5% in the eplerenone 50–100 mg group (Fig. 5).

Subgroup analyses were carried out based on the antihypertensive effect of esaxerenone on sitting BP when stratified by key patient demographics at baseline (Supplementary Table 2). Regardless of patients’ baseline SBP/DBP, grade of hypertension, PAC, PRA, eGFRcreat, serum K^+^ levels, or the presence or absence of diabetes mellitus, there was an antihypertensive effect across all subgroups.

### Safety analysis

Safety analysis showed there were no marked differences in the incidence of AEs observed between patients taking placebo (46.0% [40/87]), esaxerenone 1.25 mg/day (30.1% [25/83]), esaxerenone 2.5 mg/day (40.5% [34/84]), esaxerenone 5 mg/day (36.4% [32/88]), or eplerenone 50–100 mg/day (36.9% [31/84]) (Table 2). All AEs were determined to be either mild or moderate in severity. The most commonly observed AEs, with an incidence of ≥ 3.0% in any treatment group, were nasopharyngitis, upper respiratory tract inflammation, pharyngitis, headache, blood K^+^ increased, blood uric acid increased, blood triglycerides increased, eGFRcreat decreased, back pain, blood creatine phosphokinase increased, and musculoskeletal stiffness. In addition, no AEs occurred that were considered related to sex hormones.

**Table 2:** Adverse events (safety analysis set)

	Placebo *n* = 87	Esaxerenone 1.25 mg/day *n* = 83	Esaxerenone 2.5 mg/day *n* = 84	Esaxerenone 5 mg/day *n* = 88	Eplerenone 50–100 mg/day *n* = 84	All *N* = 426
Any adverse event	40 (46.0)	25 (30.1)	34 (40.5)	32 (36.4)	31 (36.9)	162 (38.0)
Any serious adverse event	2 (2.3)	1 (1.2)	0 (0.0)	0 (0.0)	0 (0.0)	3 (0.7)
Any drug-related adverse event	8 (9.2)	8 (9.6)	7 (8.3)	12 (13.6)	7 (8.3)	42 (9.9)
Any drug-related serious adverse event	0 (0.0)	0 (0.0)	0 (0.0)	0 (0.0)	0 (0.0)	0 (0.0)
Number of patients who are discontinued from the study due to drug-related TEAE	0 (0.0)	0 (0.0)	0 (0.0)	0 (0.0)	1 (1.2)	1 (0.2)
Number of patients who discontinued from the study due to hyperkalemia	0 (0.0)	0 (0.0)	0 (0.0)	0 (0.0)	0 (0.0)	0 (0.0)
Adverse events occurring in ≥3% patients for any group						
Nasopharyngitis	7 (8.0)	4 (4.8)	6 (7.1)	6 (6.8)	7 (8.3)	30 (7.0)
Upper respiratory tract inflammation	4 (4.6)	3 (3.6)	4 (4.8)	5 (5.7)	2 (2.4)	18 (4.2)
Pharyngitis	2 (2.3)	3 (3.6)	5 (6.0)	1 (1.1)	2 (2.4)	13 (3.1)
Headache	2 (2.3)	1 (1.2)	6 (7.1)	0 (0.0)	2 (2.4)	11 (2.6)
Back pain	3 (3.4)	1 (1.2)	1 (1.2)	1 (1.1)	0 (0.0)	6 (1.4)
Musculoskeletal stiffness	0 (0.0)	0 (0.0)	3 (3.6)	0 (0.0)	0 (0.0)	3 (0.7)
Blood creatine phosphokinase increased	3 (3.4)	0 (0.0)	1 (1.2)	0 (0.0)	0 (0.0)	4 (0.9)
Blood K^+^ increased	2 (2.3)	0 (0.0)	3 (3.6)	3 (3.4)	1 (1.2)	9 (2.1)
Blood uric acid increased	1 (1.1)	2 (2.4)	1 (1.2)	3 (3.4)	1 (1.2)	8 (1.9)
Blood triglycerides increased	0 (0.0)	2 (2.4)	2 (2.4)	0 (0.0)	3 (3.6)	7 (1.6)
eGFRcreat decreased	1 (1.1)	3 (3.6)	0 (0.0)	3 (3.4)	0 (0.0)	7 (1.6)

Data are presented as *n* (%). System Organ Classes and Preferred Terms coded using MedDRA/J version 18.0. Percentages calculated using the number of subjects in the column heading as the denominator

*TEAE* treatment-emergent adverse event, *GFR* glomerular filtration rate

Serious AEs occurred in three patients during the treatment period, but only one of these patients (emergency hypertension) was from an esaxerenone treatment group (1.25 mg/day). Although this patient was withdrawn from the study, a causal relationship with the study drug was ruled out. However, one patient was withdrawn from the eplerenone group due to a drug-related AE (diarrhea). No AEs were clinically significant, and no notable changes were observed in vital signs or body weight.

The change from baseline of serum K^+^ increased according to the dose of esaxerenone administered. Serum K^+^ increased to its highest value at weeks 1 and 2, and then reached steady state with a slight decrease over the course of the study (Figures S1 and S2). Hyperkalemia predefined as a serum K^+^ level of ≥ 6.0 mEq/L or ≥ 5.5 mEq/L on two consecutive measurements was observed in one patient treated with esaxerenone 5 mg/day (serum K^+^: 4.4 mEq/L at baseline and 6.0 mEq/L measured once at week 12), however, this promptly recovered to 4.7 mEq/L on the next day. No patients were withdrawn from the study due to increased serum K^+^ levels.

The mean (SD) changes from baseline in eGFRcreat at week 12 in the 1.25, 2.5, and 5 mg/day esaxerenone groups were −2.31 (6.85), −3.69 (7.98), and −6.36 (8.08) mL/min/1.73 m^2^, respectively. In comparison, the mean (SD) changes from baseline in eGFRcreat at week 12 for the placebo and eplerenone groups were 0.06 (6.05) and −2.11 (6.35) mL/min/1.73 m^2^, respectively.

### Pharmacokinetic analysis

Plasma esaxerenone concentration (C_trough_) levels generally increased in proportion with increasing esaxerenone dose and were similar at weeks 4 and 12. At week 4, the mean (SD) esaxerenone concentration levels were 8.61 (2.97), 16.39 (6.68), and 32.77 (17.6) ng/mL in the 1.25, 2.5, and 5 mg/day groups, respectively. At week 12, the mean (SD) esaxerenone concentrations levels were 8.67 (2.87), 15.72 (6.74), and 32.74 (15.5) ng/mL in the 1.25, 2.5, and 5 mg/day groups, respectively.

### Analysis of PAC and PRA

PAC and PRA did not change in the placebo group; however, they increased in proportion with increasing esaxerenone dose. The mean percent (95% CI) changes in PAC values from baseline until week 12 were 0.0% (−8.0 to 8.6), 18.3% (8.8 to 28.5), 30.3% (20.1 to 41.3), 35.5% (25.2 to 46.6), and 19.5% (10.2 to 29.5) in the placebo group, the esaxerenone 1.25, 2.5, and 5 mg/day groups, and the eplerenone group, respectively. The mean percent (95% CI) changes in PRA from baseline until week 12 were −5.5% (−18.9 to 10.1), 27.4% (9.2 to 48.7), 44.4% (24.2 to 67.8), 119.6% (90.0 to 153.7), and 54.5% (33.3 to 79.1) in the placebo group, the esaxerenone 1.25, 2.5, and 5 mg/day groups, and the eplerenone group, respectively.

## Discussion

This multicenter study evaluated the antihypertensive effect and safety of esaxerenone, a novel non-steroidal MR blocker. The aim was to determine the optimal dose that would lower BP in Japanese patients with essential hypertension. We demonstrated a dose–response relationship in sitting and 24-h BPs, after 12 weeks of esaxerenone treatment.

Efficacy was evidenced by significant decreases in sitting BP at esaxerenone dosages of 2.5 and 5 mg/day compared with placebo. There was also a significant difference in 24-h BP for all dosages of esaxerenone (1.25, 2.5, and 5 mg/day) compared with placebo. In comparison, the antihypertensive effect of eplerenone was extrapolated to be between the data sets in the esaxerenone 2.5 and 5 mg/day groups, which suggest that esaxerenone at 2.5–5 mg/day has a similar antihypertensive effect to the clinical dosages of eplerenone 50–100 mg/day.

Previous non-clinical studies have shown that esaxerenone has no agonistic or antagonistic effects on glucocorticoid, progesterone, or androgen receptors [24, 25]. The present clinical study therefore supports this conclusion as evidenced by the absence of sex hormone-related AEs.

In this study, there were no clear differences in the risk of increased serum K^+^ levels when compared with placebo. Although the change in serum K^+^ generally increased in proportion with esaxerenone dose and reached a maximum value at week 1 or week 2 of treatment, this increase did not continue through to week 12. The mean (SD) baseline serum K^+^ level for all patients was 4.11 (0.29) and these levels remained stable throughout the study with maximum mean (SD) differences of 0.21 (0.31), 0.27 (0.26), and 0.33 (0.31) mEq/L for the 1.25, 2.5, and 5 mg/day esaxerenone groups, respectively. In most patients, serum K^+^ levels did not reach ≥5.5 mEq/L. Hyperkalemia was only detected in one patient in the esaxerenone 5 mg/day group, however, this was transient and this patient recovered without further treatment. Hyperkalemia, a clinically relevant condition, is often a dose–response side effect of aldosterone antagonists such as spironolactone and eplerenone for the treatment of hypertension [27–30].

In the present study, esaxerenone plasma concentration increased in proportion with dose and was shown to be stable based on the similar values at week 4 and week 12. As esaxerenone dose increased, the RAAS hormone measurements represented by PAC and PRA also increased in proportion, which is indicative of an MR antagonistic effect after oral administration of esaxerenone for 12 weeks. These changes in RAAS hormones were almost identical to that observed in the previous 6-week exploratory study (not published).

In the subgroup analysis, there were reductions in sitting BP even in patients with diabetes. There were also reductions in sitting BP (SBP/DBP) across a broad range of patient demographics. Analysis of the mean change in sitting BP from baseline to the end of the study showed that differences in the baseline hypertension grade, PAC, PRA, eGFRcreat levels and serum K^+^ levels had no clear effect on BP-lowering effects of esaxerenone therapy.

The present findings indicate that 2.5 and 5 mg/day esaxerenone are optimal dosages based on the efficacy and safety data presented. The perceived limitations of this study include the small sample size, short observation period (12 weeks), and open-label nature of the comparator eplerenone. However, a randomized, double-blind, long-term phase 3 study has been completed recently to further investigate the safety and efficacy of esaxerenone therapy in essential hypertension patients. In addition, the safety and efficacy profiles of esaxerenone therapy in other patient populations, such as those affected by CKD and type 2 diabetes mellitus with albuminuria, have been assessed in several phase 3 studies that have been completed recently.

Esaxerenone is a novel non-steroidal MR blocker that has a dose-dependent antihypertensive effect on sitting SBP/DBP and 24-h BP. Esaxerenone showed good efficacy and safety profiles in Japanese essential hypertensive patients, and no obvious safety concerns, including hyperkalemia, were observed across all doses of esaxerenone. In conclusion, esaxerenone 2.5 and 5 mg/day are both considered optimum dosages for the treatment of essential hypertension.

### Summary Table

#### What is known about the topic?

- Essential hypertension and end-organ damage are leading risk factors for morbidity and mortality throughout the world.
- Current guidelines recommend combination therapies that have complementary mechanisms to reduce blood pressure. Mineralocorticoid receptor (MR) antagonists are recommended for the treatment of resistant hypertension.
- An existing MR antagonist, spironolactone, has significant treatment-emergent side effects, which affect compliance rates. Eplerenone has an improved safety profile but may have less potent antihypertensive efficacy as compared with spironolactone.

#### What this study adds

- Esaxerenone is a novel non-steroidal MR blocker that has a dose-dependent antihypertensive effect compared with placebo.
- Esaxerenone has a good safety profile and shows no adverse events related to sex hormones.
- This study determined the optimal doses of esaxerenone for lowering blood pressure in Japanese patients with essential hypertension.

## Supplementary information

Supplementary Fig.1

Supplementary Fig.2

Supplementary table 1

Supplementary Table 2

Supplementary Information
