# Supplementary material for: Efficacy and safety of esaxerenone (CS-3150) for the treatment of essential hypertension: a phase 2 randomized, placebo-controlled, double-blind study
Source: J Hum Hypertens. 2019 May 21;33(7):542–51. doi: 10.1038/s41371-019-0207-x (PMC6760614; doi:10.1038/s41371-019-0207-x)
Supplement: Supplementary file 1 — Supplementary Fig.1 [file 41371_2019_207_MOESM1_ESM.pptx]

## Slide 1
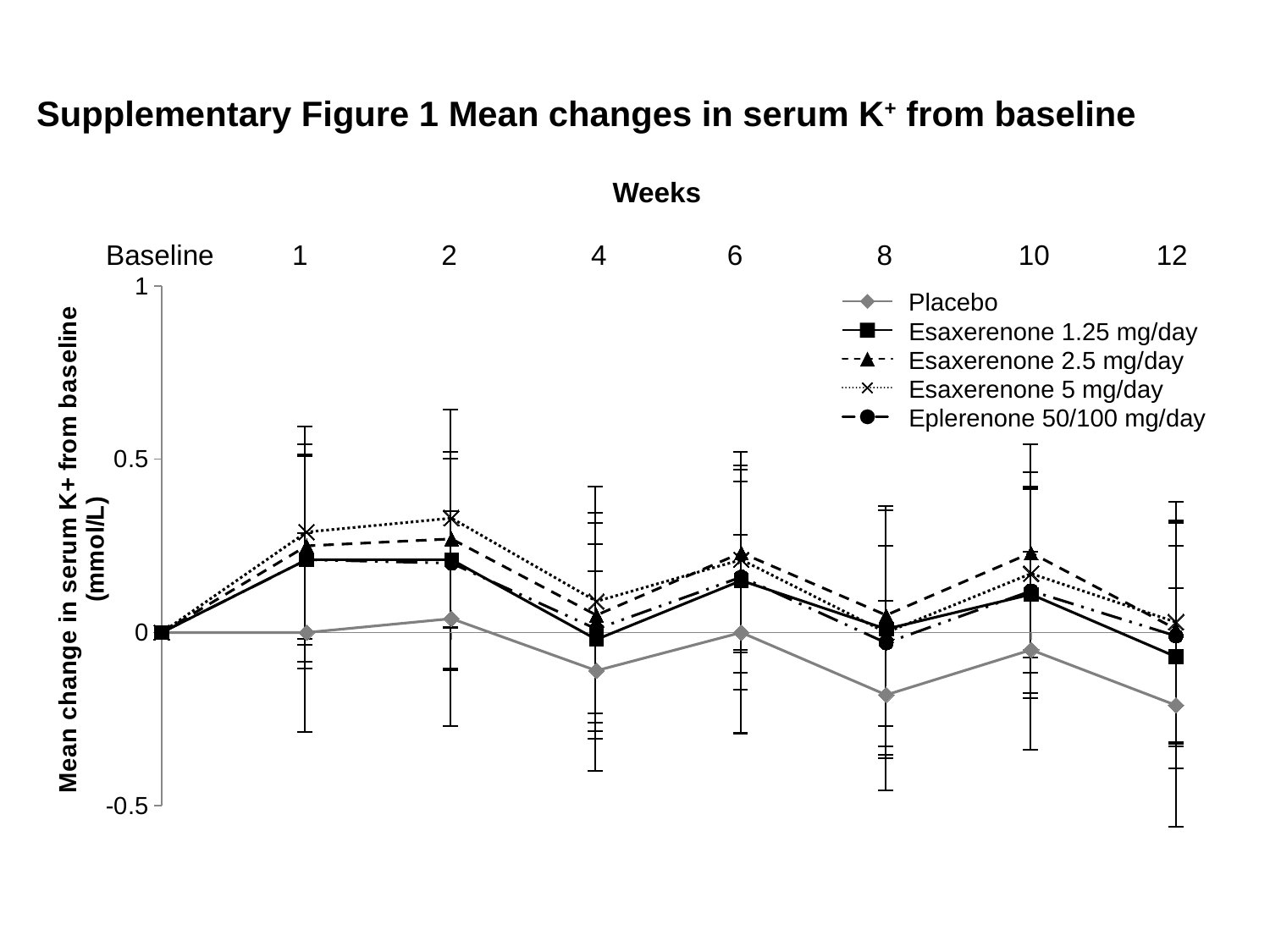

Supplementary Figure 1 Mean changes in serum K+ from baseline
Weeks
### Chart
| Category | | | | | |
|---|---|---|---|---|---|Baseline
	1	2	4	6	8	10	12
Placebo
Esaxerenone 1.25 mg/day
Esaxerenone 2.5 mg/day
Esaxerenone 5 mg/day
Eplerenone 50/100 mg/day
