# Supplementary material for: Efficacy and safety of esaxerenone (CS-3150) for the treatment of essential hypertension: a phase 2 randomized, placebo-controlled, double-blind study
Source: J Hum Hypertens. 2019 May 21;33(7):542–51. doi: 10.1038/s41371-019-0207-x (PMC6760614; doi:10.1038/s41371-019-0207-x)
Supplement: Supplementary file 2 — Supplementary Fig.2 [file 41371_2019_207_MOESM2_ESM.pptx]

## Slide 1
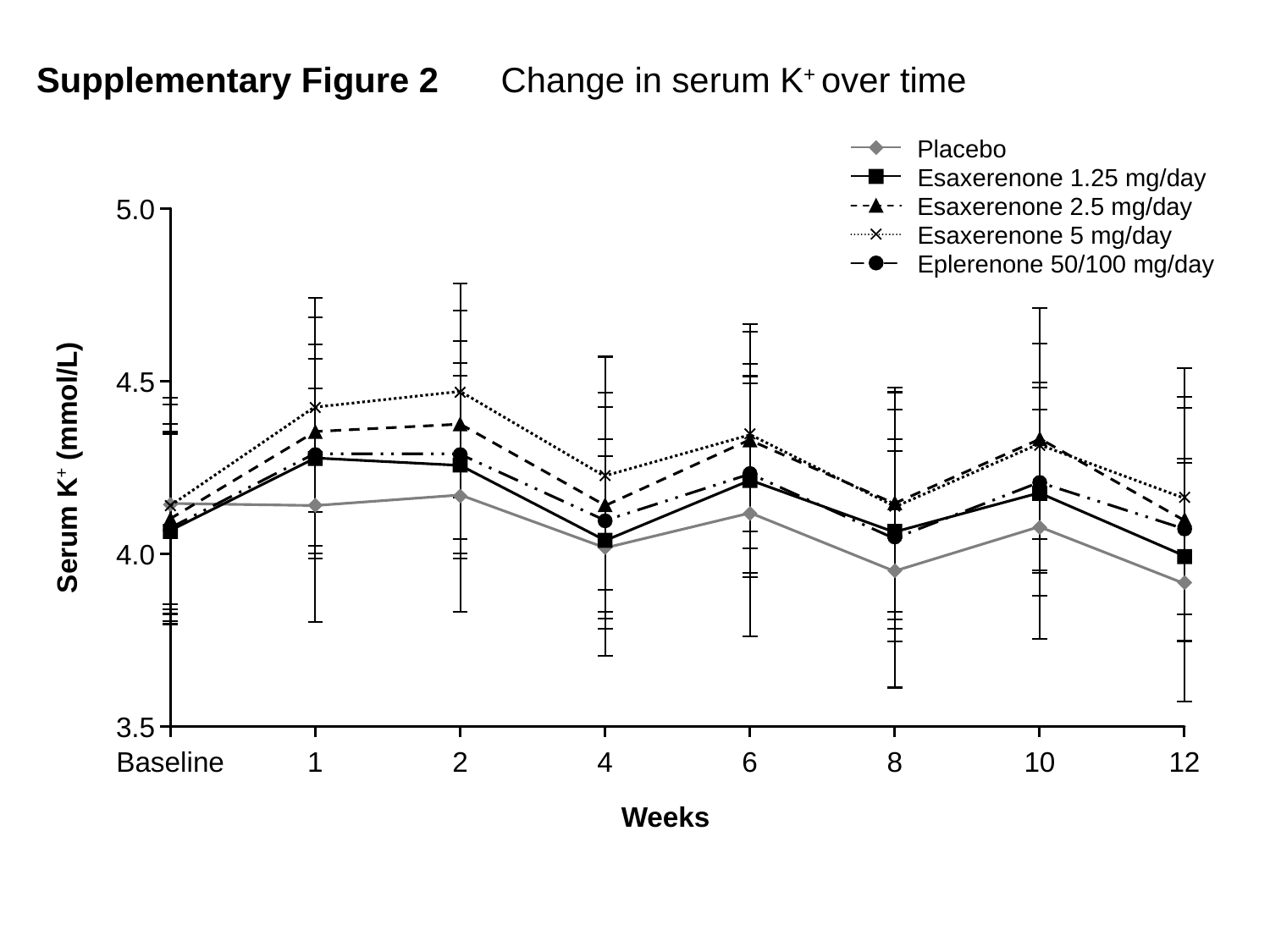

Supplementary Figure 2 　Change in serum K+ over time
Placebo
Esaxerenone 1.25 mg/day
Esaxerenone 2.5 mg/day
Esaxerenone 5 mg/day
Eplerenone 50/100 mg/day
5.0
4.5
Serum K+ (mmol/L)
4.0
3.5
Baseline
1
2
4
6
8
10
12
Weeks
