# Supplementary material for: Efficacy and safety of esaxerenone (CS-3150) for the treatment of essential hypertension: a phase 2 randomized, placebo-controlled, double-blind study
Source: J Hum Hypertens. 2019 May 21;33(7):542–51. doi: 10.1038/s41371-019-0207-x (PMC6760614; doi:10.1038/s41371-019-0207-x)
Supplement: Supplementary file 3 — Supplementary table 1 [file 41371_2019_207_MOESM3_ESM.docx]

**Supplementary Table 1**

**Summary**

Table showing the least squares mean changes from baseline in ambulatory blood pressure in the morning (06:00–08:59), daytime (07:00–21:59), and nocturnal (22:00–06:59) segments.

**Supplementary Table 1.** Summary of change in morning, daytime, and nocturnal ambulatory blood pressure

| ABPM | Placebo *n*=75 | Esaxerenone 1.25 mg/day *n*=77 | Esaxerenone 2.5 mg/day *n*=77 | Esaxerenone 5 mg/day *n*=83 | Eplerenone 50–100 mg/day *n*=82 |
| --- | --- | --- | --- | --- | --- |
| Morning |  |  |  |  |  |
| SBP (mmHg) | 3.8 (0.2, 7.4) | −4.2 (−7.7, −0.7) | −5.4 (−8.9, −1.9) | −16.9 (−20.3, −13.5) | −11.1 (−14.5, −7.7) |
| DBP (mmHg) | 2.4 (0.3, 4.5) | −2.0 (−4.0, 0.1) | −2.0 (−4.1, 0.1) | −8.4 (−10.4, -6.4) | −4.6 (−6.6, −2.5) |
| Daytime |  |  |  |  |  |
| SBP (mmHg) | −2.2 (−5.5, 1.2) | −8.4 (−11.7, −5.2) | −10.4 (−13.7, −7.1) | −19.1 (−22.2, −15.9) | −12.5 (−15.6, −9.3) |
| DBP (mmHg) | −0.9 (−2.7, 1.0) | −3.3 (−5.1, −1.4) | −4.9 (−6.7, −3.1) | −9.9 (−11.7, −8.2) | −6.5 (−8.3, −4.7) |
| Nocturnal |  |  |  |  |  |
| SBP (mmHg) | 2.7 (−0.6, 6.0) | −2.6 (−5.8, 0.7) | −8.0 (−11.3, −4.7) | −14.1 (−17.3, −11.0) | −9.2 (−12.4, −6.1) |
| DBP (mmHg) | 2.0 (0.3, 3.8) | −0.2 (−2.0, 1.5) | −3.6 (−5.4, −1.9) | −6.6 (−8.3, −4.9) | −4.5 (−6.2, −2.8) |

Least squares mean change (95% confidence intervals) in blood pressure from baseline using an analysis of covariance model with treatment group as a factor and baseline blood pressure level as a covariate. The time segments were as follow: morning (06:00–08:59), daytime (07:00–21:59), and nocturnal (22:00–06:59).

ABPM, ambulatory blood pressure monitoring; DBP, diastolic blood pressure; SBP, systolic blood pressure
