# Supplementary material for: Efficacy and safety of esaxerenone (CS-3150) for the treatment of essential hypertension: a phase 2 randomized, placebo-controlled, double-blind study
Source: J Hum Hypertens. 2019 May 21;33(7):542–51. doi: 10.1038/s41371-019-0207-x (PMC6760614; doi:10.1038/s41371-019-0207-x)
Supplement: Supplementary file 4 — Supplementary Table 2 [file 41371_2019_207_MOESM4_ESM.docx]

**Supplementary Table 2**

**Summary**

Table showing the least squares mean (95% CI) change in sitting BP (SBP/DBP) as stratified by baseline SBP/DBP, hypertension grade, PAC, PRA, eGFRcreat, serum K^+^ levels, and the presence or absence of diabetes mellitus

**Supplementary Table 2.** Subgroup analysis of sitting blood pressure stratified by patient demographics at baseline

| Least square mean change from baseline |  | Placebo | Esaxerenone 1.25 mg/day | Esaxerenone 2.5 mg/day | Esaxerenone 5 mg/day | Eplerenone 50–100 mg/day |
| --- | --- | --- | --- | --- | --- | --- |
| Sitting BP (SBP)  (95% CI) | Number of patients | 85 | 82 | 84 | 88 | 84 |
|  | Baseline SBP <160 mmHg | −5.8  (−8.6, −3.0) | −10.5  (−13.3, −7.7) | −12.3  (−15.0, −9.7) | −19.5  (−22.3, −16.8) | −14.9  (−17.8, −12.0) |
|  | Baseline SBP ≥160 mmHg | −9.3  (−13.9, −4.7) | −10.9  (−15.8, −6.0) | −17.8  (−22.9, −12.8) | −22.5  (−27.0, −18.0) | −21.2  (−25.6, −16.7) |
|  | Baseline DBP <100 mmHg | −8.1  (−10.9, −5.3) | −13.0  (−16.0, −10.1) | −15.5  (−18.6, −12.3) | −21.6  (−24.5, −18.8) | −19.1  (−22.2, −15.9) |
|  | Baseline DBP ≥100 mmHg | −3.4  (−8.1, 1.2) | −5.3  (−9.6, −1.0) | −12.6  (−16.4, −8.9) | −18.2  (−22.4, −14.1) | −15.9  (−19.7, −12.2) |
|  | Hypertension Grade I^†^ | −6.7  (−9.7, −3.8) | −12.9  (−16.0, −9.8) | −13.6  (−16.9, −10.4) | −19.4  (−22.5, −16.4) | −16.0  (−19.3, −12.7) |
|  | Hypertension Grade II^†^ | −7.2  (−10.9, −3.4) | −8.7  (−12.4, −4.9) | −15.1  (−18.6, −11.5) | −21.4  (−25.0, −17.8) | −18.6  (−22.0, −15.1) |
|  | PAC <120 pg/mL | −6.2  (−9.6, −2.8) | −11.6  (−14.8, −8.4) | −13.2  (−16.3, −10.0) | −21.5  (−24.5, −18.6) | −19.4  (−22.7, −16.1) |
|  | PAC ≥120 pg/mL | −8.3  (−11.9, −4.8) | −8.3  (−12.3, −4.3) | −17.0  (−21.1, −13.0) | −17.9  (−22.0, −13.7) | −14.0  (−17.7, −10.4) |
|  | PRA <1.0 ng/mL/h | −7.8  (−11.3, −4.3) | −10.6  (−14.0, −7.3) | −15.1  (−18.6, −11.5) | −21.8  (−24.9, −18.7) | −17.8  (−21.4, −14.3) |
|  | PRA ≥1.0 ng/mL/h | −6.3  (−9.8, −2.8) | −10.6  (−14.6, −6.7) | −13.4  (−16.9, −10.0) | −17.5  (−21.5, −13.4) | −16.6  (−20.0, −13.1) |
|  | eGFRcreat <90 mL/min/1.73m^2^ | −6.2  (−8.8, −3.5) | −9.7  (−12.4, −7.0) | −14.8  (−17.5, −12.1) | −21.0  (−23.5, −18.4) | −18.8  (−21.5, −16.0) |
|  | eGFRcreat ≥90 mL/min/1.73m^2^ | −11.9  (−18.1, −5.8) | −16.6  (−23.1, −10.2) | −12.3  (−18.1, −6.4) | −17.6  (−24.8, −10.5) | −12.6  (−17.8, −7.5) |
|  | Serum K^+^ <4.5 mEq/L | −7.1  (−9.7, −4.5) | −10.9  (−13.5, −8.4) | −14.8  (−17.4, −12.3) | −20.2  (−22.7, −17.6) | −16.3  (−18.9, −13.7) |
|  | Serum K^+^ ≥4.5 mEq/L | −5.5  (−13.0, 1.9) | −7.7  (−16.7, 1.3) | −10.9  (−20.0, −1.8) | −24.1  (−31.8, −16.5) | −26.3  (−34.4, −18.3) |
|  | Presence of diabetes | −16.3  (−25.1, −7.4) | −5.3  (−13.2, 2.5) | −14.3  (−23.5, −5.1) | −19.9  (−28.1, −11.7) | −18.8  (−24.7. −12.9) |
|  | Absence of diabetes | −6.0  (−8.5, −3.4) | −11.5  (−14.1, −8.9) | −14.3  (−16.9, −11.8) | −20.7  (−23.2, −18.2) | −17.0  (−19.8, −14.2) |
| Sitting BP (DBP)  (95% CI) | Number of patients | 85 | 82 | 84 | 88 | 84 |
|  | Baseline SBP <160 mmHg | −3.5  (−5.3, −1.8) | −5.4  (−7.2, −3.7) | −7.5  (−9.2, −5.8) | −10.5  (−12.2, −8.8) | −7.3  (−9.2, −5.5) |
|  | Baseline SBP ≥160 mmHg | −4.2  (−6.5, −1.8) | −4.1  (−6.7, −1.6) | −8.0  (−10.6, −5.4) | −10.3  (−12.6, −8.0) | −10.1  (−12.4, −7.8) |
|  | Baseline DBP <100 mmHg | −4.4  (−6.0, −2.7) | −6.1  (−7.9, −4.3) | −8.0  (−9.9, −6.0) | −10.5  (−12.2, −8.7) | −8.9  (−10.8, −7.0) |
|  | Baseline DBP ≥100 mmHg | −2.7  (−5.3, 0.0) | −2.7  (−5.2, −0.3) | −7.0  (−9.1, −4.8) | −10.4  (−12.7, −8.0) | −7.6  (−9.7, −5.5) |
|  | Hypertension Grade I^†^ | −3.8  (−5.8, −1.9) | −6.3  (−8.3, −4.2) | −7.7  (−9.8, −5.5) | −10.1  (−12.1, −8.1) | −7.6  (−9.8, −5.4) |
|  | Hypertension Grade II^†^ | −3.7  (−5.8, −1.7) | −3.9  (−5.9, −1.9) | −7.7  (−9.6, −5.8) | −10.7  (−12.6, −8.8) | −9.0  (−10.9, −7.1) |
|  | PAC <120 pg/mL | −3.6  (−5.6, −1.7) | −5.2  (−7.1, −3.4) | −7.1  (−8.9, −5.3) | −11.0  (−12.8, −9.3) | −9.0  (−10.9, −7.1) |
|  | PAC ≥120 pg/mL | −4.0  (−6.0, −1.9) | −4.5  (−6.8, −2.2) | −9.2  (−11.6, −6.9) | −8.5  (−10.8, −6.1) | −7.4  (−9.4, −5.3) |
|  | PRA <1.0 ng/mL/hr | −4.2  (−6.2, −2.1) | −5.0  (−6.9, −3.1) | −7.6  (−9.7, −5.6) | −10.7  (−12.5, −8.9) | −8.4  (−10.4, −6.3) |
|  | PRA ≥1.0 ng/mL/hr | −3.1  (−5.1, −1.2) | −5.1  (−7.3, −2.9) | −8.0  (−9.9, −6.1) | −9.3  (−11.6, −7.1) | −8.4  (−10.3, −6.4) |
|  | eGFRcreat <90 mL/min/1.73m^2^ | −3.1  (−4.6, −1.6) | −4.5  (−6.1, −3.0) | −7.8  (−9.3, −6.2) | −10.5  (−11.9, −9.0) | −9.1  (−10.7, −7.5) |
|  | eGFRcreat ≥90 mL/min/1.73m^2^ | −7.9  (−11.6, −4.1) | −7.7  (−11.5, −3.9) | −7.4  (−10.9, −3.8) | −9.1  (−13.5, −4.8) | −5.9  (−9.0, −2.7) |
|  | Serum K^+^ <4.5 mEq/L | −3.7  (−5.3, −2.2) | −5.4  (−6.9, −3.9) | −7.7  (−9.2, −6.2) | −10.3  (−11.7, −8.8) | −8.0  (−9.6, −6.5) |
|  | Serum K^+^ ≥4.5 mEq/L | −4.2  (−8.0, −0.5) | −1.0  (−5.9, 3.9) | −6.8  (−11.3, −2.2) | −11.6  (−15.5, −7.7) | −11.9  (−16.0, −7.7) |
|  | Presence of diabetes | −7.7  (−12.4, −3.1) | −3.7  (−7.9, 0.6) | −7.0  (−11.9, −2.0) | −8.7  (−13.1, −4.3) | −8.7  (−11.8, −5.6) |
|  | Absence of diabetes | −3.3  (−4.8, −1.8) | −5.2  (−6.8, −3.7) | −7.7  (−9.2, −6.2) | −10.6  (−12.1, −9.2) | −8.4  (−10.1, −6.8) |

^†^Hypertension grades as defined by Japanese Society of Hypertension 2014 guidelines.

BP, blood pressure; DBP, diastolic blood pressure; eGFRcreat, creatinine-adjusted estimated glomerular filtration rate; PAC, plasma aldosterone concentration; PRA, plasma renin activity; SBP, systolic blood pressure
