# Supplementary material for: Efficacy and safety of esaxerenone (CS-3150) for the treatment of essential hypertension: a phase 2 randomized, placebo-controlled, double-blind study
Source: J Hum Hypertens. 2019 May 21;33(7):542–51. doi: 10.1038/s41371-019-0207-x (PMC6760614; doi:10.1038/s41371-019-0207-x)
Supplement: Supplementary file 5 — Supplementary Information [file 41371_2019_207_MOESM5_ESM.docx]

**Supplementary Information**

**Summary**

Additional details of inclusion and exclusion criteria, and the rationale for sample size calculation are provided.

**Inclusion criteria**

In addition to the inclusion criteria described in the main text, patients with grade I or grade II essential hypertension had to satisfy both of the following criteria: arithmetic mean blood pressure (BP) at the last two time points in the observation period of systolic BP (SBP) ≥140 mmHg and <180 mmHg and diastolic BP (DBP) ≥90 mmHg and <110 mmHg; and BP measured during the last two time points in the observation period stable within 30 mmHg for SBP and within 15 mmHg for DBP. Furthermore, after week 3 of the observation period, a 24-hour BP (as determined by ambulatory BP monitoring) of SBP ≥130 mmHg and DBP ≥80 mmHg was required.

**Exclusion criteria**

In addition to the exclusion criteria described in the main text, patients with cerebral or cardiovascular disease who met the following criteria were excluded: occurrence of acute coronary syndrome in the six months before written informed consent, percutaneous coronary intervention or coronary artery bypass graft surgery in the six months before written informed consent, revascularization of the carotid or peripheral artery in the six months before written informed consent, occurrence of cerebral stroke or transient ischemic attack in the year before written informed consent, presence of a New York Heart Association class III or IV heart failure, presence of life-threatening intractable valve stenosis, or the presence of life-threatening intractable ventricular arrhythmia. Patients who meet the following criteria were also excluded: presence of orthostatic hypotension or with a history of orthostatic hypotension; under treatment for malignancies; presence of diabetes mellitus with albuminuria and a urine albumin-to-creatinine ratio of ≥ 30 mg/g·CRE that was measured twice during the observation period; aspartate transaminase or alanine transaminase ≥ 100 IU/L; HbA1c (measured using the National Glycohemoglobin Standardization Program) ≥ 8.4%; serious hepatic disease (e.g. hepatic failure and hepatic cirrhosis); previously hospitalized for hyperkalemia in the year before written informed consent; use of any prohibited drug that violates the criteria for prohibited concomitant drugs or who are likely to need one during the study period; a history of a significant adverse drug reaction (e.g. electrolyte abnormality and acute renal failure) to spironolactone or eplerenone; have previously received esaxerenone; a history of severe drug allergy; patients whose day–night life is reversed (e.g. night shift work); have received any other study drug in the 12 weeks before written informed consent; women who are or may be pregnant, breastfeeding women, and female patients (including female partners of male patients) who plan to become pregnant during the study period; and those patients judged by the investigator or sub investigator to be ineligible for participation in the study.

**Sample size calculation**

This study had a planned target sample size of 400, based on observing a change in the primary endpoint from baseline to the end of treatment. The following assumptions were made in reference to the results of a phase IIa preliminary study of esaxerenone in essential hypertension (unpublished data): that the change in sitting BP would be −6.0/−3.0 mmHg in the placebo group and −14.0/−7.0 mmHg in the 1.25 mg esaxerenone group; that the estimated standard deviation for change in sitting BP would be 12.0/7.0 mmHg; and that a dose response could be anticipated for the relationship between sitting BP and esaxerenone dose.

Based on these assumptions, the probability of a Type I error was set at 5% (two-tailed), and a sample size of 49 (SBP) or 66 (DBP) patients per group would be required to detect superiority in paired comparisons of SBP and DBP between the placebo group and esaxerenone doses of ≥1.25 mg with a 90% power. Assuming a drop-out rate of 5%, and in consideration of the lowered power of test for multiplicity using a fixed-sequence method, the target sample size was set at 80 patients per group.
